# An 8‐channel receive array for improved ^31^P MRSI of the whole brain at 3T

**DOI:** 10.1002/mrm.27736

**Published:** 2019-03-21

**Authors:** Mark J. van Uden, Tom H. Peeters, Anne Rijpma, Christopher T. Rodgers, Arend Heerschap, Tom W. J. Scheenen

**Affiliations:** ^1^ Department of Radiology and Nuclear Medicine Radboud university medical center Nijmegen The Netherlands; ^2^ Department of Geriatric Medicine Radboud university medical center Nijmegen The Netherlands; ^3^ Radboudumc Alzheimer Center, Donders Institute for Brain, Cognition and Behaviour Radboud university medical center Nijmegen The Netherlands; ^4^ Wolfson Brain Imaging Centre University of Cambridge Cambridge United Kingdom; ^5^ Erwin L. Hahn Institute University Hospital Duisburg‐Essen Essen Germany

**Keywords:** ^1^H‐decoupling, 3 Tesla, ^31^P MR spectroscopic imaging, brain, RF array coil

## Abstract

**Purpose:**

To demonstrate a ^1^H/^31^P whole human brain volume coil configuration for 3 Tesla with separate ^31^P transmit and receive components that maintains ^1^H MRS performance and delivers optimal ^31^P MRSI with ^1^H decoupling.

**Methods:**

We developed an 8‐channel ^31^P receive array coil covering the head to be used as an insert for a commercial double‐tuned ^1^H/^31^P birdcage transmit‐receive coil. This retains the possibility of using low‐power rectangular pulses for ^1^H‐decoupled 3D ^31^P MRSI (nominal resolution 17.6 cm^3^; acquisition duration 13 min) but increases the SNR with the receive sensitivity of ^31^P surface coils. The performance of the combined coil setup was evaluated by measuring ^1^H and ^31^P SNR with and without the ^31^P receive array and by assessing the effect of the receive array on the transmit efficiencies of the birdcage coil.

**Results:**

Compared to the birdcage coil alone, the ^31^P insert in combination with the birdcage achieved an average ^31^P SNR gain of 1.4 ± 0.4 in a center partition of the brain. The insert did not cause losses in ^1^H MRS performance and transmit efficiency, whereas for ^31^P approximately 20% more power was needed to achieve the same γB1.

**Conclusion:**

The new coil configuration allows ^1^H MRSI and optimal ^1^H‐decoupled 3D ^31^P MRSI, with increased SNR of the human brain without patient repositioning, for clinical and research purposes at 3 Tesla.

## 1 INTRODUCTION

In vivo phosphorus MRS (^31^P MRS) of the brain allows to noninvasively measure brain metabolites that are linked to the energy and phospholipid metabolism. It is used to study several neurological diseases and other pathologies, such as cancer, in which metabolite levels are altered.^1^, ^2^

Although the unique value of ^31^P MRS in the noninvasive examination of brain diseases has already been demonstrated for this purpose many years ago (e.g., Refs. 3,4), it is much less used than ^1^H MRS,^5^ mainly because of a lower sensitivity and the need for additional hardware. To obtain localized ^31^P MR spectra of the human brain, it is common to employ a birdcage type of coil for transmit/receive with either a single or multivoxel MRSI pulse sequence.^6^, ^7^ For anatomical guidance by MRI and B_0_‐shimming, it is required that the ^31^P coil is integrated with a ^1^H radiofrequency (RF) element, which can also be used for ^1^H‐decoupling, inducing the nuclear Overhauser effect and ^1^H MRS.

To overcome that low SNR hampers quantification of metabolite peaks, the lower sensitivity of ^31^P MRS compared to ^1^H MRS can be compensated by prolonging acquisition times, enlarging voxels, and/or using a higher magnetic field. However, sensitivity can also be enhanced by reducing the size of the receiving RF coils, in particular at the more superficial regions of the brain.^8^ Multiple small receive elements arranged in a 2D or 3D array will result in an SNR increase while maintaining a large FOV. Receive arrays are particularly attractive in combination with a homogeneous transmit coil. This enables uniform excitation across the FOV by excitation with low power radiofrequency pulses instead of adiabatic pulses with large power deposition in the tissue, the latter resulting in a high specific absorption rate (SAR). This leaves room in ^31^P experiments to apply RF‐pulses for ^1^H‐decoupling and/or inducing nuclear Overhauser effect for increased spectral resolution and SNR, respectively.

Because 3 Tesla (T) systems are widely available in the clinic, many sites could take advantage of an improved coil design rather than switching to expensive ultrahigh field (≥7T) systems. Although X‐nuclei phased array coils are not new,^9^, ^10^, ^11^ to our knowledge a ^31^P phased array coil insert to be used in combination with a commercially available ^1^H/^31^P birdcage coil has not been designed for the brain at 3T.

In this work, we aimed to maximize SNR and spectral resolution, and thereby the applicability of ^31^P MRS at 3T, by developing a dedicated 8‐channel ^31^P receive head‐array coil that can be used in combination with a ^1^H/^31^P birdcage transmit‐receive coil to enable ^1^H‐decoupling and nuclear Overhauser effect. We acquired in vivo ^31^P spectra of the brain and compared the performance of the coil setup with and without the ^31^P 8‐channel insert.

## 2 METHODS

### 2.1 Coil design

An 8‐channel ^31^P receive head‐array coil was designed to be combined with a commercially available quadrature Tx/Rx ^1^H/^31^P birdcage coil (RAPID Biomedical GmbH, Rimpar, Germany). Both coils were actively detunable at the ^31^P frequency. To this end, the commercial coil had to be adjusted by the manufacturer. For ^1^H applications, the birdcage coil was always used to both transmit and receive.

**Figure 1:**
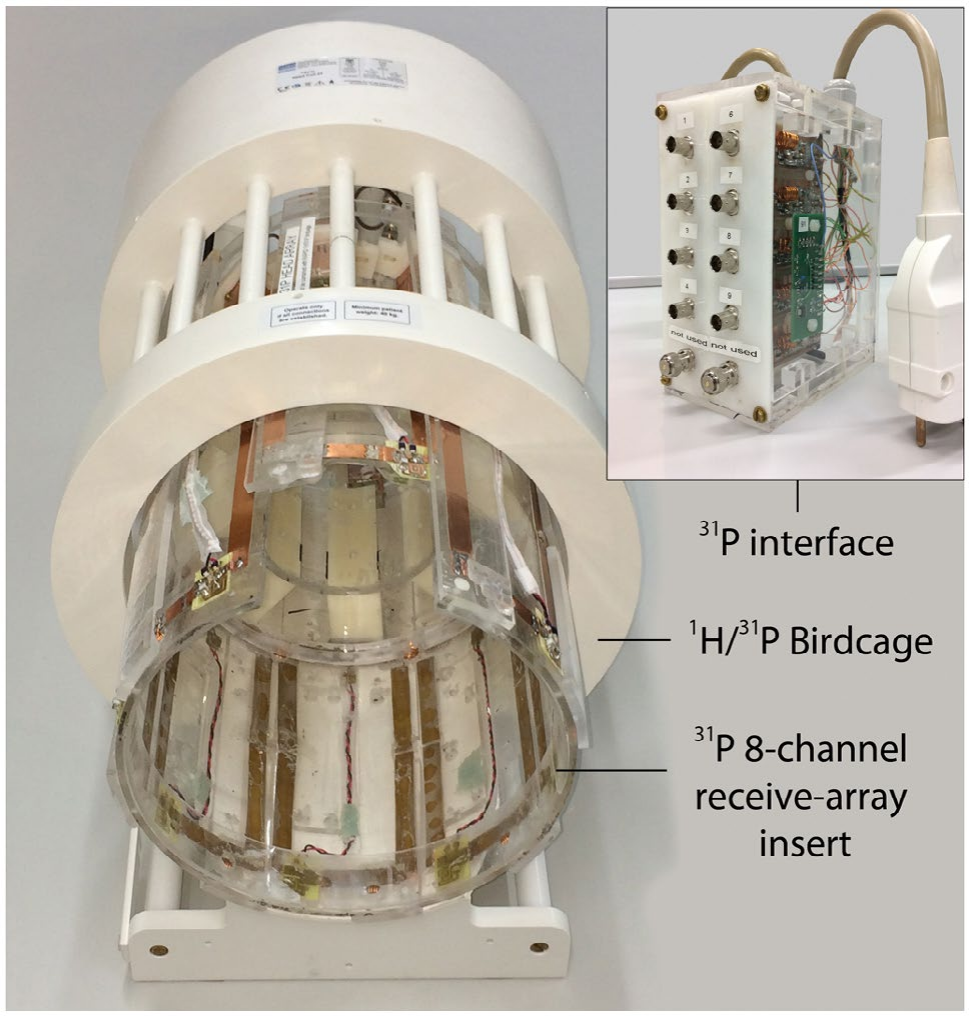
Overview of the combined coil configuration. The ^31^P 8‐channel head‐array coil is positioned inside a commercially available, detunable ^1^H/^31^P birdcage. The insert is connected to the MR system via a custom‐built ^31^P interface. The frontal element at the side of the patient’s face is smaller than the other 7 elements to maximize the subject’s comfort

The elements of the head array were constructed using 12.7 mm‐wide copper tape with a thickness of 35.6 µm on a Plexiglas cylinder with an outer diameter of 24.5 cm. The dimensions of the elements were 100 × 200 mm, except for the frontal element (100 × 100 mm) to create space for the patient’s nose and view, herewith improving patient comfort (Figure 1). The loaded and unloaded quality factors of the elements and element coupling (S21) were measured on the workbench with a vector network analyzer (R&HZVL3, Rohde & Schwarz, Munich, Germany). Adjacent elements, separated by a gap of 0.5 cm, were isolated from each other by means of capacitive decoupling.^12^ Furthermore, all elements were isolated by preamp decoupling.^13^

Two active ^31^P detuning circuits, as well as 2 ^1^H traps^14^ per element, prevented coupling between the transmit field and the receive elements, which could otherwise have produced hotspots with high local SAR. The cables of the receive array were connected to an interface box, which was intentionally positioned behind the setup to keep distance between the transmit coil and receive circuits. To prevent ^1^H power loss, 4 cables were equipped with additional ^1^H cable traps.

### 2.2 Interface box

**Figure 2:**
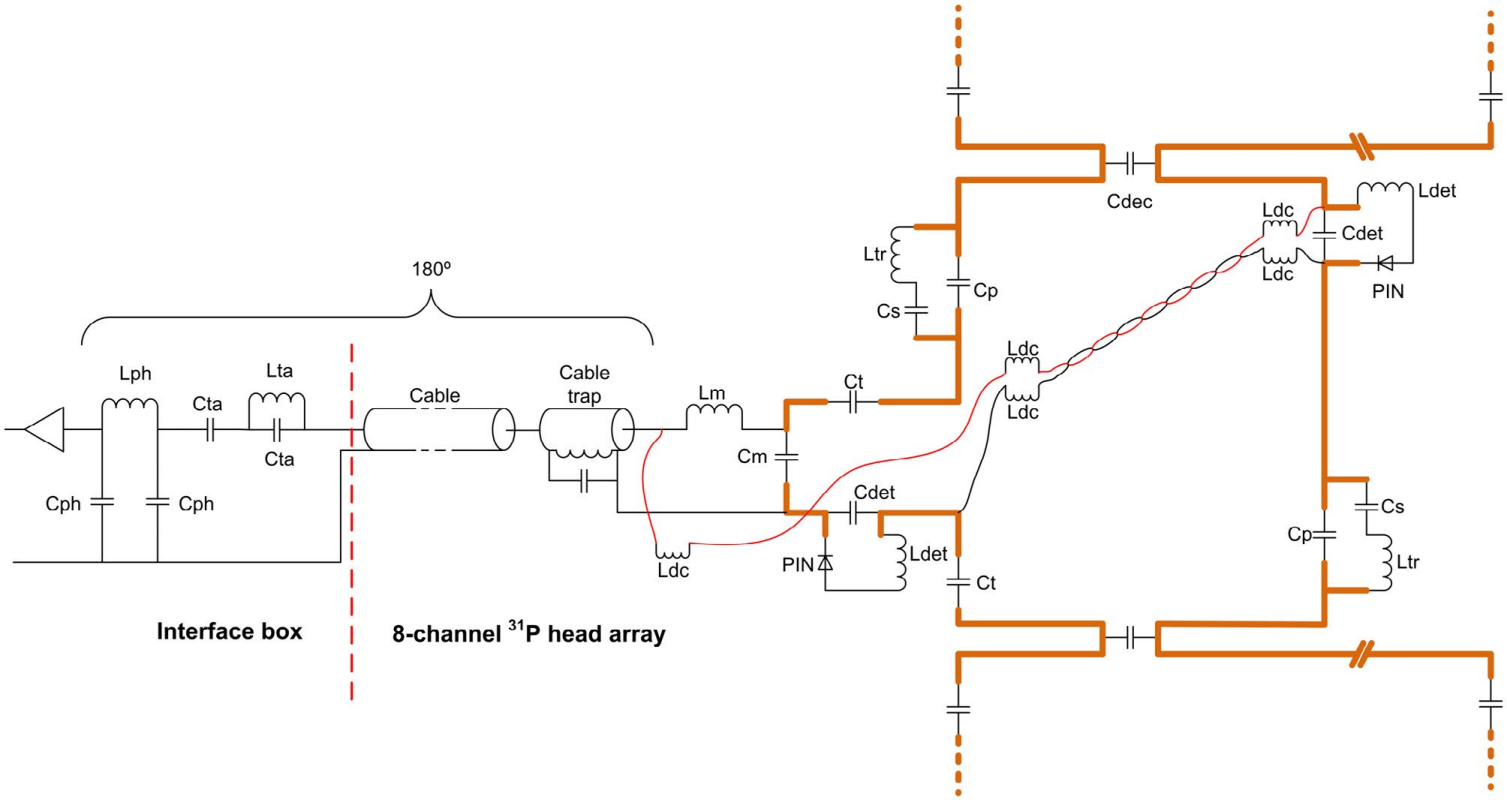
Diagram of ^31^P head array electric circuit. Detailed overview of all components of the anterior element, the connection to the interface, and the components of the interface electronics. The component functions are indicated as: decoupling capacitor (Cdec), tuning capacitors (Ct), matching circuit (Cm, Lm), RF‐block inductors (Ldc), ^31^P active detuning circuit (Ldet, Cdet), improved ^1^H trap circuits (Cs, Cp, Ltr ), PIN‐diodes (PIN), ^1^H tank circuit (Lta, Cta), and the phase shifter (Lph, Cph)

The interface box contains low‐input impedance preamplifiers, phase shifters, and ^1^H tank circuits. The matching circuit used to introduce preamplifier decoupling, as proposed by Roemer et al.,^13^ requires the low input impedance from the preamplifier at its ports. This was achieved by a 180‐degree phase shift between the input from the preamplifier and the matching circuit. The phase shift was a cumulative effect of the cable, cable traps, and phase shifters (Figure 2). Note that no attempt was made to adjust the relative phase of the receive elements in hardware. This is because the MR system receives and digitizes all channels separately and then in software deduces the appropriate weights for optimal signal combination using the whitened singular value decomposition algorithm.^15^

The functionality of this circuitry setup was confirmed on the workbench by means of S21 measurements using 2 isolated pickup probes. ^1^H tank circuits prevented the ^31^P spectra from contamination with spurious signals during ^1^H‐decoupling. Coil files were adjusted for automated coil detection and proper coil control by the MR system.

### 2.3 Measurements

Because the presence of the ^31^P head array coil in the ^1^H/^31^P birdcage coil might influence the transmit fields locally and therefore could exceed SAR limits, we performed temperature measurements on phantoms placed in the combination of both coils with maximum transmit power on the birdcage coil. The commercial birdcage coil is CE‐approved with characterized global SAR levels; thus, we verified that insertion of the phased array coil would not result in local SAR hotspots exceeding these approved levels. The load of a human head was mimicked by a cylindrical phantom (diameter = 16 cm, height = 29 cm) containing phosphoric acid. Temperature measurements were performed with an optical probe (Luxtron,

LumaSense Technologies, Santa Clara, CA) positioned in a small gel phantom with 3 wt% agar and 0.5 wt% NaCl while transmitting at maximum RF power of either ^1^H or ^31^P frequency. Because there is no heat transfer through convection or perfusion in the gel phantom, this represents a worst‐case situation compared to measurements in vivo. The gel phantom was positioned on spots that were suspected to carry locally high electrical fields, such as ^31^P detune circuits, ^1^H traps, and capacitors. Furthermore, the coil was checked by feeling by hand if any heating occurred on the housing of the insert.

The performance of the birdcage alone and the combined setup were tested with 3 healthy volunteers (2 female, aged: 28.3 ± 3.2 y) on a 3T MR‐system (Magnetom Trio, Siemens Healthcare, Erlangen, Germany). The ^31^P transmit efficiencies were compared by means of a slice selective pulse‐acquire experiment (TR = 15 s) covering the brain through assessment of the voltage that corresponds to a γB_1_ of 500 Hz, reflected by a maximized PCr magnitude signal. ^1^H transmit efficiencies were determined automatically by the MR system. To determine the noise correlation between the array elements, a single‐slice ^31^P gradient‐echo image was acquired while transmit power was set to 0.

A 3D MRSI FID sequence with a WALTZ4 (Wideband Alternating phase Low‐power Technique for Zero residue splitting) ^1^H‐decoupling scheme was used to acquire ^31^P MR spectroscopic images of the whole brain for SNR comparisons between both coil setups. TR was 2000 ms. For excitation, a pulse with a duration of 500 µs and a flip angle of 40° (Ernst angle, assuming a maximum T_1_ of 7500 ms) were applied. The dead time between pulse and acquisition was 100 μs. For 3D ^31^P MRSI, the FOV was set to 260 × 260 × 260 mm^3^ applying Hamming‐weighted k‐space sampling, averaging 4 FIDs of 1024 data points around the center of a 10 × 10 × 10 k‐space matrix. The voxel size defined as 64% of the point spread function area was approximately 40 cm^3^.^16^ The measurement time was 13 min and 8 s. WALTZ4 ^1^H‐decoupling (γB_1_ = 250 Hz) was turned on during the first 256 ms (50%) of the acquisition window.

For ^1^H SNR comparison, ^1^H MR spectra of the anterior part of the occipital lobe were acquired with a single voxel PRESS sequence using chemical shift selective water suppression.^17^, ^18^ TR/TE were set to 3000/30 ms to measure a 20 × 20 × 16 mm^3^ voxel with a flip angle of 90° (64 averages).

All volunteer studies were conducted with approval of the institutional review board of the Radboud university medical center, The Netherlands.

### 2.4 Postprocessing

The amplitude and relative phase of the signal of each array coil element depend on the magnetization and position of the excited volume with respect to the receive elements. Because these parameters vary per array coil element, we used the whitened singular value decomposition algorithm for signal combination.^15^

Before Fourier transform, the in vivo 3D ^31^P MRSI data set was interpolated to a 16 × 16 × 16 matrix with a nominal voxel size of 16.25 × 16.25 × 16.25 mm^3^. The SNR performance of the birdcage alone and the combination with the phased‐array insert were evaluated in the center transversal partition of the 3D dataset in all volunteers. SNR was calculated as the ratio of the integral of the PCr peak, as fitted by Metabolite Report in syngo (work‐in‐progress package; Siemens Healthineers, Erlangen, Germany), divided by the SD of the noise. Noise SD was calculated from a signal‐free portion of the spectrum. An average noise correlation matrix was calculated from the noise in the ^31^P gradient‐echo images.

To evaluate the ^1^H performance of the birdcage with and without the ^31^P array inserted, the SNRs of all single voxel ^1^H spectra, acquired from the anterior part of the occipital lobe, were analyzed with LCModel version 6.3‐0C software using a simulated basis set of 24 metabolites.^19^ SNR is defined as the maximum of the NAA signal at 2.01 ppm in the baseline‐corrected spectrum divided by 2 times the RMS of the residuals.^20^

## 3 RESULTS

**Figure 3:**
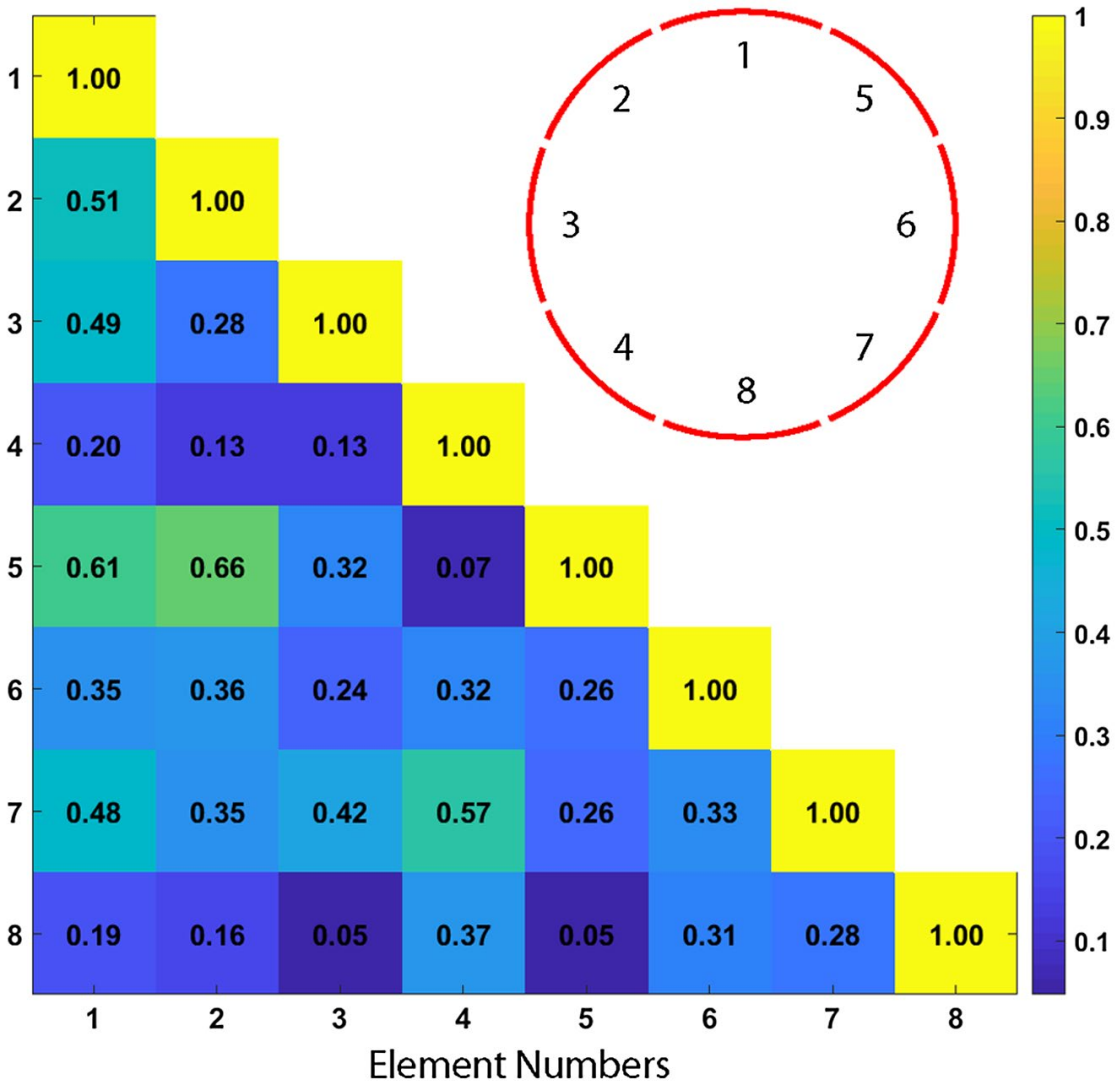
Noise correlation map of the ^31^P head‐array elements. The noise correlation map was calculated from gradient‐echo data. On the vertical and the horizontal axis are the element numbers. The red gapped circle represents the element numbering of the head array as seen from the feet side

To assess the performance of the ^31^P receive head array, we determined the Q‐factor ratio Q_UL_/Q_L_ for all individual array elements. The values for the Q ratio ranged from 1.4 to 2, depending on the head‐to‐element distance. Next, we determined the port‐to‐port (S21) decoupling, which ranged from −10 dB to −15 dB between neighboring elements and from −8.5 dB to −22.6 dB for non‐neighboring elements. The calculated noise correlation matrix showed variations in noise correlation between both neighboring and non‐neighboring elements (Figure 3).

To check whether SAR limits would be exceeded when transmitting with the birdcage coil in the combined coil configuration, temperature was measured at maximum transmit power of either the ^1^H or ^31^P frequency. This did not result in a temperature increase in the gel phantom, from which we conclude that the International Electrotechnical Commission limits were not exceeded. The temperature of the housing directly above the detuning circuits and cable traps was elevated but did not exceed 41°C. Based on these 2 results, the same SAR limits as used for the CE‐approved ^1^H/^31^P birdcage coil could be applied to the combined setup.

The presence of the array coil did not cause any losses in ^1^H transmit efficiency, whereas for the ^31^P channel a 20% loss was observed, likely due to the close proximity of the array electronics to the conductors of the birdcage. The SNR of ^1^H and ^31^P signals for the whole brain, as obtained from the NAA (2.01 ppm) and PCr signals, respectively, was studied with the ^1^H/^31^P birdcage coil only and with a combination of this coil and the ^31^P receive head‐array insert.

**Figure 4:**
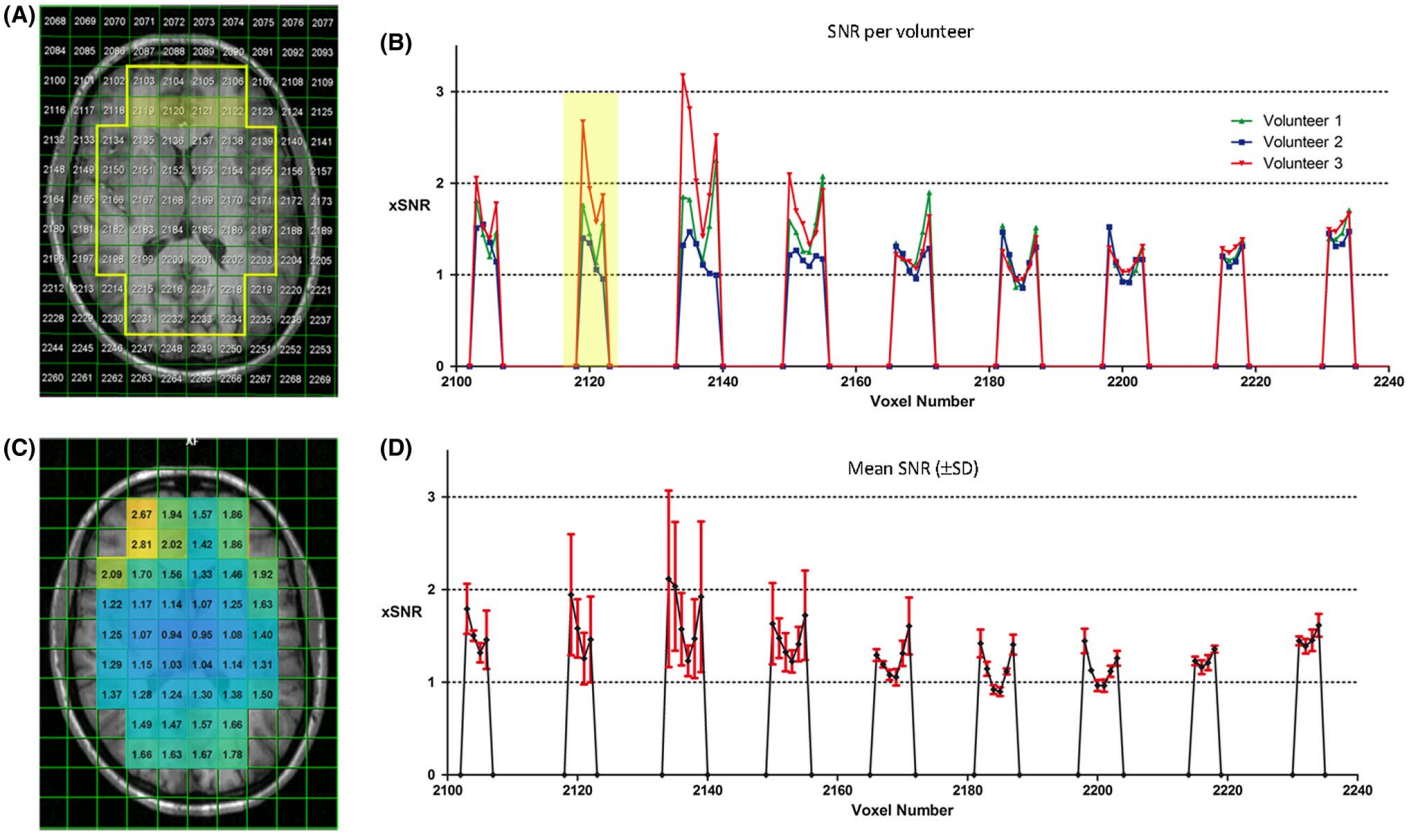
SNR gain by the ^31^P head array. A transversal image of the brain with a region of interest (yellow line) and an overlay of corresponding MRSI voxel numbers (A). The SNR in the region of interest is represented row‐wise and per volunteer in (B). Voxels of the second row are marked with a yellow bar. Line colors represent the different volunteers. The average SNR of all volunteers is projected on the brain image in (C) and is presented row‐wise (± SD) in (D)

The ^1^H/^31^P birdcage coil has a flat ^31^P receive profile, and the combined probe showed a ^31^P profile with a radially increasing sensitivity from the center of the head toward the array elements. The SNR gain of the PCr peak due to the insertion of the ^31^P array coil varied from 0.9 in the center up to a factor of 3.2 in the anterior part of the brain. The average SNR gain (± SD) of all selected voxels was 1.4 ± 0.4 (Figure 4). The average linewidth (FWHM) of PCr in the marked voxels of Figure 4A, which include frontal brain areas, was 12.4 ± 7.5 Hz for the birdcage‐only and 14.4 ± 7.4 Hz in the combined setup.

To assess the ^1^H MRS sensitivity for both coil configurations, we used the SNR value of the NAA methyl peak at 2.01 ppm. For the birdcage, the ^1^H SNR of a volume in the occipital lobe of the brain was 19, 20, and 17 for volunteers 1, 2, and 3, respectively. After inserting the ^31^P head array, the SNRs were 20, 18, and 19. Ergo, the SNRs of ^1^H MR spectra were not influenced by the presence of the ^31^P array coil.

**Figure 5:**
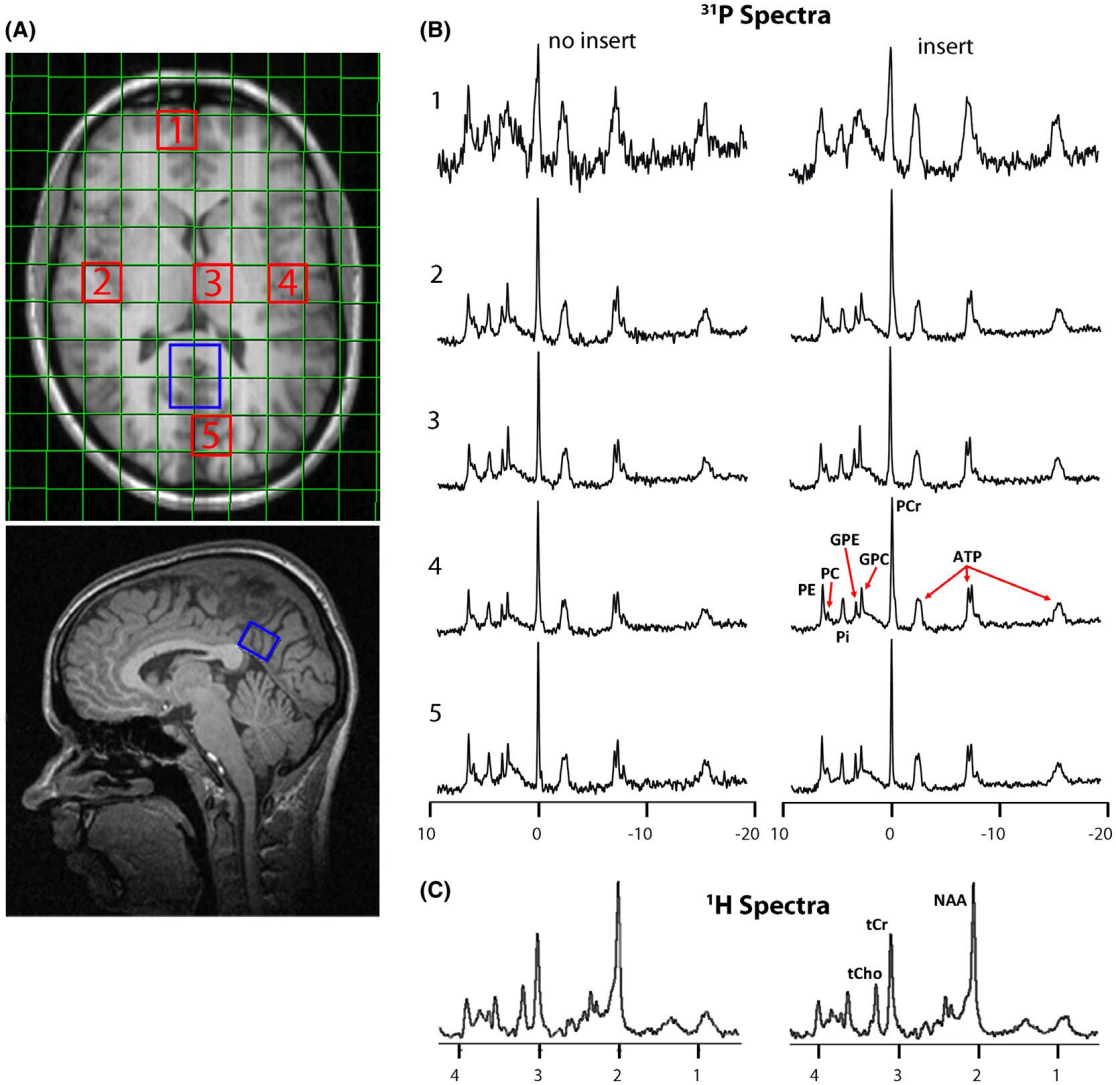
Examples of ^31^P and ^1^H spectra acquired with the birdcage only and combined with the phased array insert. Indication of ^1^H (blue) and ^31^P (red) voxel positioning in a transversal and sagittal slice of the brain (A). ^31^P (B) and ^1^H (C) spectra were acquired without (left column) and with (right column) the insert array. Both ^1^H spectra were received with the birdcage. ^31^P spectra were acquired with WALTZ4 ^1^H‐decoupling. Resolved signals of phosphoethanolamine (PE), phosphocholine (PC), inorganic phosphate (Pi), glycerophosphoethanolamine (GPE) and glycerophosphocholine (GPC), PCr, and adenosine triphosphate (ATP) are indicated in the ^31^P spectra. In the ^1^H spectrum, total Cho (tCho), total Cr (tCr), and NAA are marked

With a standard FID MRSI sequence, the resonances of phosphomonoesters, phosphodiesters, and adenosine triphosphate are not well resolved due to ^1^H‐^31^P J‐coupling. Applying ^1^H‐decoupling can remove this heteronuclear coupling and results in well‐resolved signals of phosphoethanolamine, phosphocholine, glycerophosphoethanolamine, glycerophosphocholine, and adenosine triphosphate. ^31^P signals were not affected by spurious signals or additional noise due to ^1^H‐decoupling (Figure 5).

## 4 DISCUSSION

In this work, we combined a home‐built ^31^P 8‐channel receive‐array insert with a double‐tuned ^1^H/^31^P birdcage transmit/receive coil to enable single‐session combined ^31^P and ^1^H MRSI examinations without the need of repositioning the patient in clinical routine, trials, and research at 3T.

Traditionally, ^31^P MR spectroscopy and spectroscopic imaging mostly have been performed with surface coils. Because RF transmit surface coils have an extremely inhomogeneous transmit field, the flip angles of conventional excitation pulses depend on the distance to the coil and need calibration. This can be largely overcome by adiabatic pulses.^21^ However, these pulses require high power levels, which make it a challenge not to exceed SAR limits, especially when techniques such as ^1^H‐decoupling and ^1^H‐^31^P nuclear Overhauser effect are applied. Our combined coil configuration takes advantage of separated transmit and receive elements. This allows us to generate a homogeneous transmit field with the volume ^1^H‐^31^P birdcage coil and to achieve a high sensitivity close to the array coil. For excitation, this allows us to employ rectangular pulses at lower RF power. Because shorter repetition times can be achieved with rectangular pulses, flip angle calibration can be performed fast and more averages with small flip angles acquired in the same amount of time, resulting in a higher SNR per unit time.

With the head‐array coil insert, we achieved an increase up to 3.2‐fold in SNR in superficial anterior brain areas compared to the birdcage coil alone while almost maintaining similar SNR in the center of the brain. This SNR profile is in line with other studies using an array of coils.^9^, ^22^

Noise correlation values of the array coil ranged from 0.05 up to 0.66, with a mean of 0.31 ± 0.16. This is comparable to reported values of a commercially available 8‐channel ^1^H coil^23^ but higher than reported for other ^31^P and ^1^H home‐built coils: 0.1^11^ and 0.12.^23^ The Q_UL_/Q_L _ratio of an unloaded single element is approximately halved when placed inside the birdcage. The ratio also depends on the element position in the birdcage, which implies a shared mutual resistance. Furthermore, with both the 8‐channel array and a sample present inside the birdcage, location‐dependent coupling caused extra interelement coupling of the array elements. A better decoupling between birdcage and receive elements would further improve the performance of the array. However, for safety and regulatory reasons it was not possible to make modifications to the commercial coil.

Although the maximum γB_1_ for ^31^P of the birdcage was reduced by ~20% in the presence of the array coil, the probe was still capable of generating a 90^°^ flip angle with a bandwidth of approximately 48 ppm, which is sufficient to excite all ^31^P spins relevant for in vivo applications.^24^

## 5 CONCLUSION

The combination of an 8‐channel ^31^P head array with a double‐tuned ^31^P/^1^H birdcage offers the advantages of an increase in ^31^P SNR while retaining a homogeneous transmit field on both frequencies. The losses in ^1^H performance and the ^31^P‐transmit performance of the birdcage were negligible or could be overcome. Our setup facilitates combined in vivo ^1^H and ^31^P MRSI examinations on a clinical 3T system without repositioning the subject. The proposed coil configuration in this study paves the way for human applications at 3T with more advanced sequences such as selectively refocused insensitive nuclei enhanced by polarization transfer.^25^ Another future perspective for the probe is the possibility of more efficient and informative data sampling by interleaved ^31^P and ^1^H MRS acquisitions on current state‐of‐the‐art MR‐systems.
